# Supplementary material for: Mixed twitch and tetanus electrical stimulation via belt-electrode effectively attenuates denervation-induced muscle atrophy
Source: PLoS One. 2025 Oct 15;20(10):e0334691. doi: 10.1371/journal.pone.0334691 (PMC12527172; doi:10.1371/journal.pone.0334691)
Supplement: S1 Raw Image — (PDF) [file pone.0334691.s001.pdf]

S1 Figure3 a, Western blot image

a: Phosphorylated p70S6K

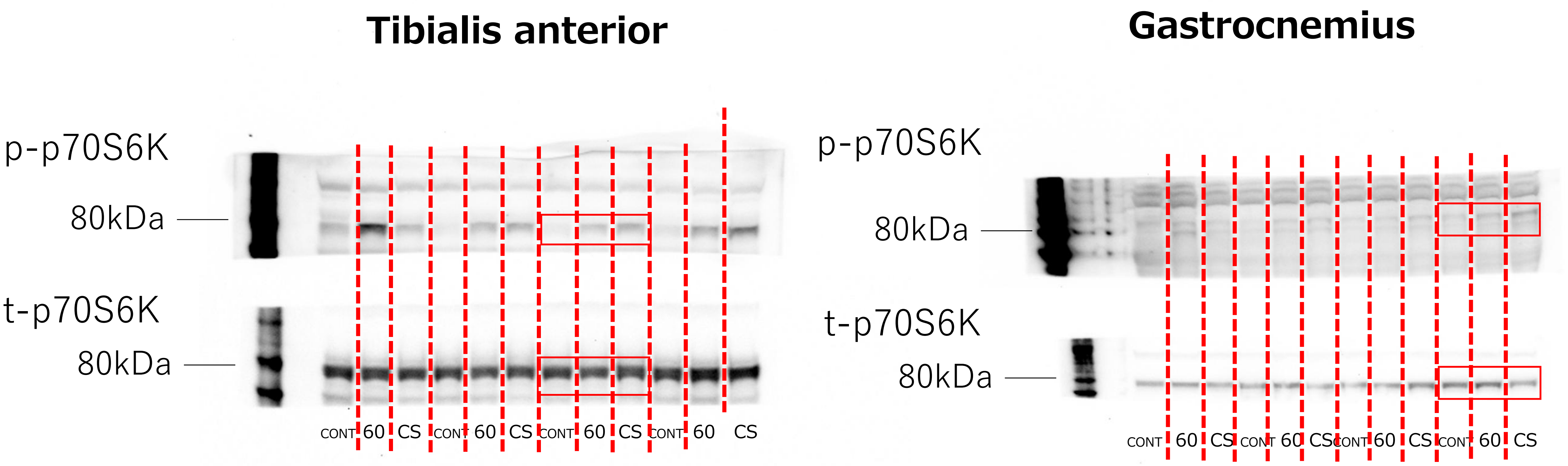

Red frame: used in the figure

The membrane was cut at the position of the target molecular weight when the antibody reaction.

S1 Figure3 a, Western blot image

b: Phosphorylated AMPK

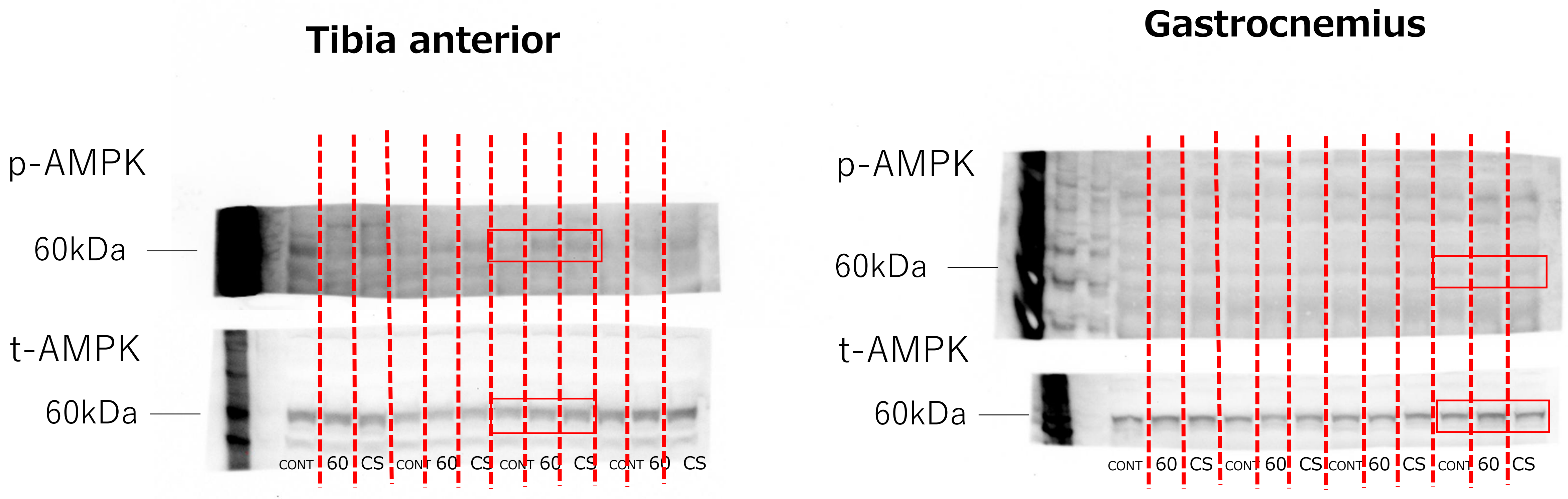

Red frame: used in the figure

The membrane was cut at the position of the target molecular weight when the antibody reaction.

Figure 5

Muscle fiber type composition ratio

Tibia anterior

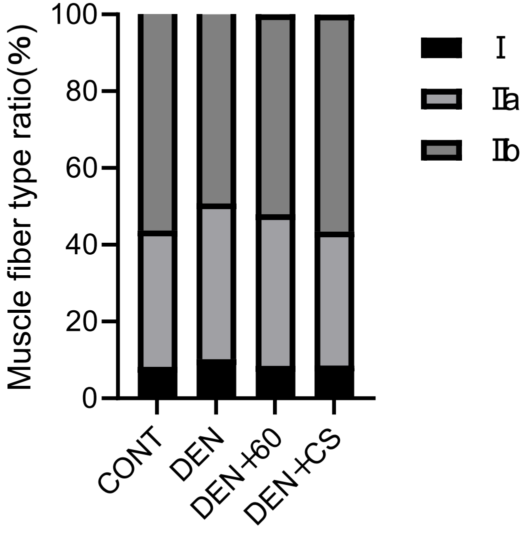

Gastrocnemius

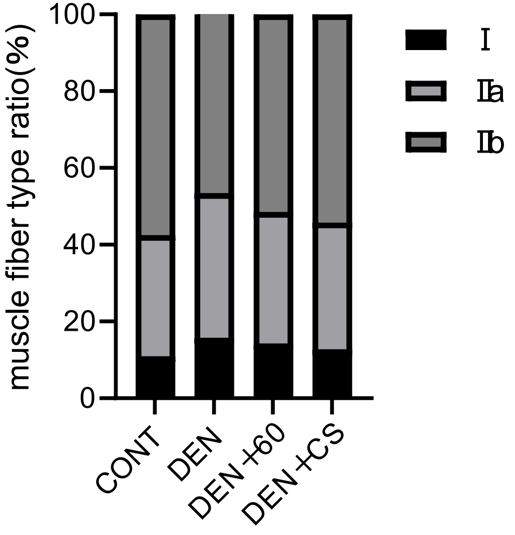

Figure 6 a western blot image

a: COXⅣ

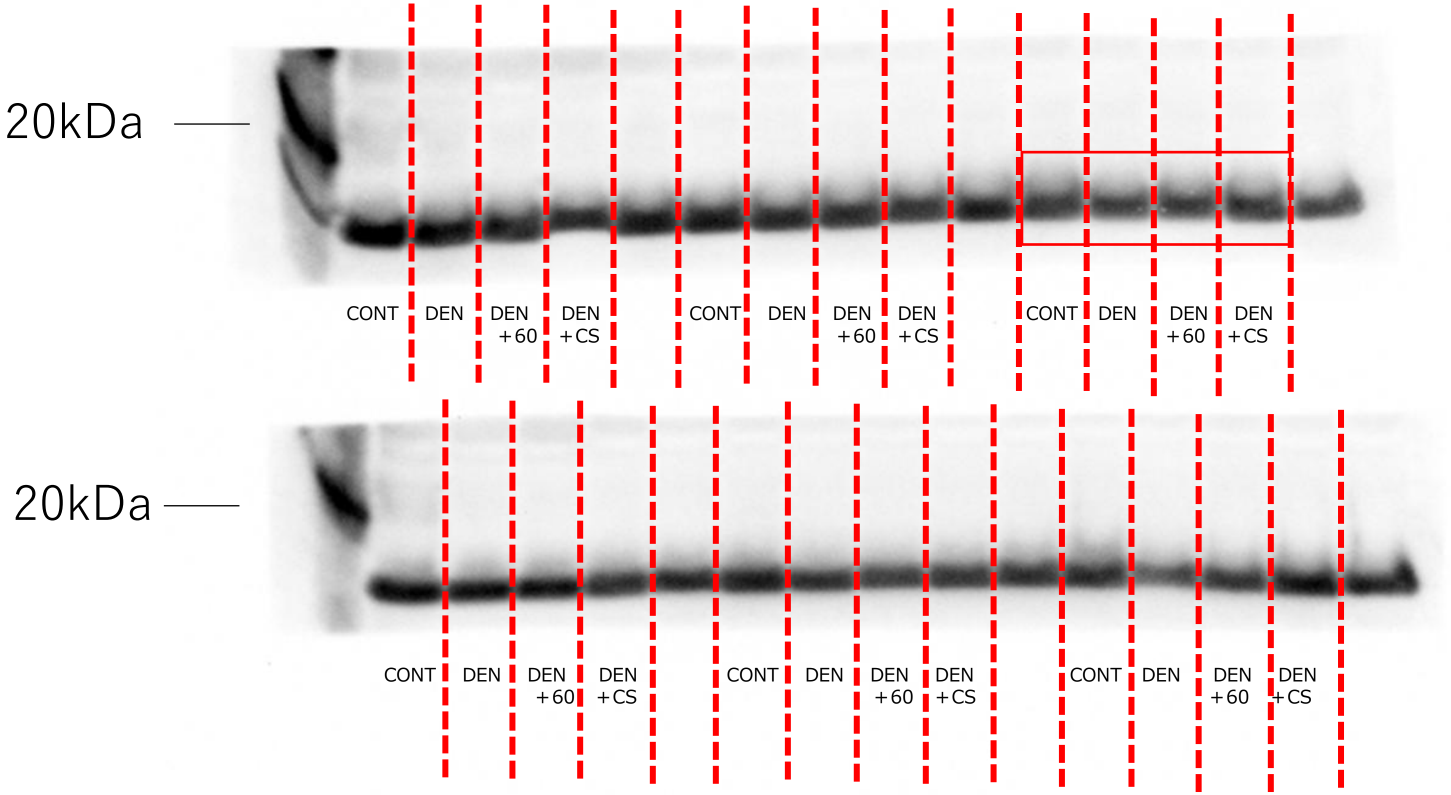

Red frame: used in the figure

The membrane was cut at the position of the target molecular weight when the antibody reaction.

Figure 6 RNA concentration of each sample used for the 18S and 28S searches

CONT	DEN	60	CS
10196.23	14664.34	14897.07	13434.36
11092.07	13842.01	16505.64	13538.21
11416.37	14440.82	16159.62	15212.93
12675.62	14365.68	15338.84	14156.52
10720.95	17643.8	15061.85	13868.99
13368.68	11946.38	16708.7	13994.38

RNA concentration (ng/uL/wet weight(g))

Figure 6 b electrophoresis image

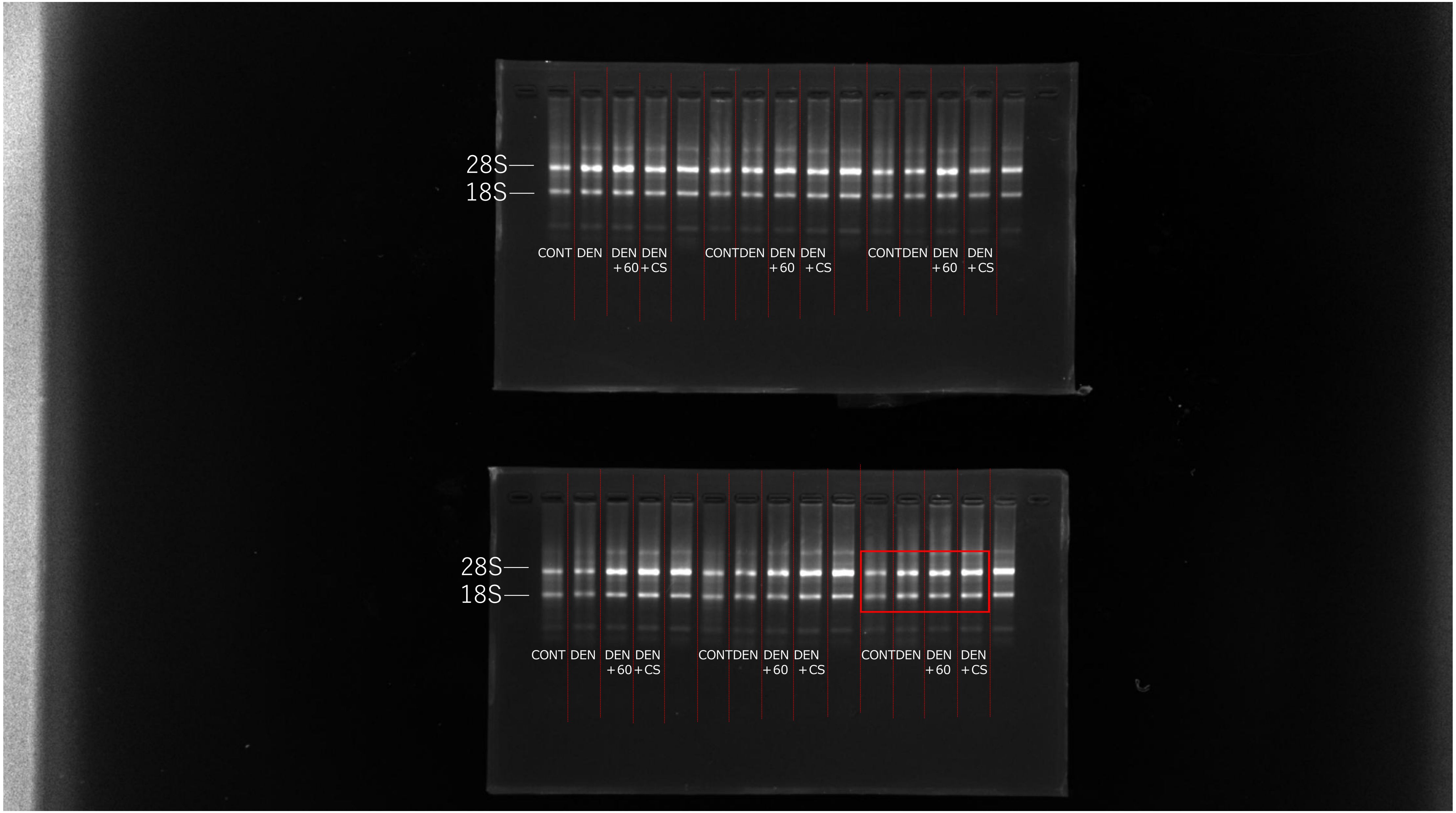

Red frame: used in the figure
