# Supplementary material for: Mixed twitch and tetanus electrical stimulation via belt-electrode effectively attenuates denervation-induced muscle atrophy
Source: PLoS One. 2025 Oct 15;20(10):e0334691. doi: 10.1371/journal.pone.0334691 (PMC12527172; doi:10.1371/journal.pone.0334691)
Supplement: Attachment — Submitted filename: PONE-D-25-28377.docx [file pone.0334691.s002.docx]

**Reviewer Comment:  PONE-D-25-28377**

1. This is a well-designed study combining twitch and tetanus stimulation to combat muscle atrophy. The idea is novel and valuable, especially for clinical applications in populations with limited mobility. However, please more clearly differentiate how your combined approach surpasses traditional methods using only tetanic stimulation, especially in terms of clinical scalability or mechanistic superiority.
2. The acute and chronic study arms are well constructed. Still, it would help to include a schematic diagram summarizing group allocation, stimulation protocols (e.g., duration, frequency, intensity), and outcome measurements.
3. Auhors shown a good evidence that mixed stimulation enhances both mitochondrial and protein synthesis markers. Still, consider adding a discussion on possible molecular pathways that explain the additive/synergistic effects.
4. While results are promising, some comparisons (e.g., between DEN+60 and DEN+CS) show only trends, not significant differences. Please clarify which results are statistically significant and which are not.
5. The figures are informative, but some (especially Western blot and CSA images) appear low-resolution. Please upload clearer images and ensure consistent formatting of figure legends.
6. Include exact p-values wherever possible and define the number of animals (n) per group for each analysis in both the text and figure legends.
7. The manuscript reads well overall, but a few sections (especially the Results and Discussion) would benefit from language polishing for clarity and flow.
8. Author mention future use in postoperative or elderly patients. It would be great to briefly explain how this method could be implemented practically in clinical or rehabilitation settings.
9. Please discuss limitations such as the short duration (7 days) of chronic stimulation and whether longer-term studies or human trials are being considered.
10. Maintain consistent terminology (e.g., use "TA" and "GAS" uniformly, and define all abbreviations on first use) to avoid confusion for non-specialist readers**.**
