# Supplementary material for: Mixed twitch and tetanus electrical stimulation via belt-electrode effectively attenuates denervation-induced muscle atrophy
Source: PLoS One. 2025 Oct 15;20(10):e0334691. doi: 10.1371/journal.pone.0334691 (PMC12527172; doi:10.1371/journal.pone.0334691)
Supplement: Attachment — Submitted filename: Response to Reviewers.docx [file pone.0334691.s005.docx]

Editor

**Comment 1**

1. Please ensure that your manuscript meets PLOS ONE's style requirements, including those for file naming.

**Reply**

Thank you for your check and reply. The PLOS ONE style requirements (including file naming requirements) were reviewed, and the manuscript was revised.

**Comment 2**

2. PLOS ONE now requires that authors provide the original uncropped and unadjusted images underlying all blot or gel results reported in a submission’s figures or Supporting Information files. This policy and the journal’s other requirements for blot/gel reporting and figure preparation are described in detail at https://journals.plos.org/plosone/s/figures#loc-blot-and-gel-reporting-requirements and https://journals.plos.org/plosone/s/figures#loc-preparing-figures-from-image-files. When you submit your revised manuscript, please ensure that your figures adhere fully to these guidelines and provide the original underlying images for all blot or gel data reported in your submission. See the following link for instructions on providing the original image data: https://journals.plos.org/plosone/s/figures#loc-original-images-for-blots-and-gels.

In your cover letter, please note whether your blot/gel image data are in Supporting Information or posted at a public data repository, provide the repository URL if relevant, and provide specific details as to which raw blot/gel images, if any, are not available. Email us at plosone@plos.org if you have any questions.

**Reply**

Thank you for the comment. During the antibody reaction, the membrane was cleaved at the desired molecular weight. Therefore, the membrane is very small.

The original, uncropped and unadjusted images are provided in supplemental information. The above explanation is provided in supplemental information.

**Comment 3**

3. PLOS requires an ORCID iD for the corresponding author in Editorial Manager on papers submitted after December 6th, 2016. Please ensure that you have an ORCID iD and that it is validated in Editorial Manager. To do this, go to ‘Update my Information’ (in the upper left-hand corner of the main menu), and click on the Fetch/Validate link next to the ORCID field. This will take you to the ORCID site and allow you to create a new iD or authenticate a pre-existing iD in Editorial Manager.

**Reply**

Thank you for the comment. I accessed the ORCID site, created a new ID, and confirmed it in the Editorial Manager.

**Comment 4**

4. To comply with PLOS ONE submissions requirements, in your Methods section, please provide additional information regarding the experiments involving animals and ensure you have included details on (1) methods of sacrifice, (2) methods of anesthesia and/or analgesia, and (3) efforts to alleviate suffering.

**Reply**

Thank you for the comment. For experiments involving animals, (1) the method of sacrifice, (2) the method of anesthesia and/or analgesia, and (3) efforts to alleviate suffering were added to the Animal section of Materials & Methods.

**Comment 5**

5. We note that the grant information you provided in the ‘Funding Information’ and ‘Financial Disclosure’ sections do not match.

When you resubmit, please ensure that you provide the correct grant numbers for the awards you received for your study in the ‘Funding Information’ section.

**Reply**

Thank you for the comment. Funding for this research was provided by HOMER ION Laboratory Co., Ltd. as joint research funding, not an award.

We have corrected inconsistencies in the grant information provided in the "Funding Information" and "Financial Disclosures" sections.

**Comment 6**

6. Thank you for stating the following financial disclosure:

[Research funding for this study was provided by HOMER ION Co., Ltd.,].

Please state what role the funders took in the study. If the funders had no role, please state: ""The funders had no role in study design, data collection and analysis, decision to publish, or preparation of the manuscript.""

If this statement is not correct you must amend it as needed.

Please include this amended Role of Funder statement in your cover letter; we will change the online submission form on your behalf.

**Reply**

Thank you for the comment. Research funding for this study was provided by HOMER ION Laboratory Co., Ltd. as a collaborative research grant.

The roles of authors who are employees of HOMER ION Laboratory Co., Ltd. in this study have been described in the cover letter.

**Comment 7**

7. Thank you for stating the following in the Competing Interests section:

[Authors with competing interests

Research funding for this study was provided by HOMER ION Co., Ltd., H.U., K.S., R.A., K.H., S.T., and M.I. are HOMER ION Co., Ltd. employees, and K.N. is a co-researcher. The other authors have no financial disclosures related to this paper.].

We note that one or more of the authors are employed by a commercial company: name of commercial company.

1. Please provide an amended Funding Statement declaring this commercial affiliation, as well as a statement regarding the Role of Funders in your study. If the funding organization did not play a role in the study design, data collection and analysis, decision to publish, or preparation of the manuscript and only provided financial support in the form of authors' salaries and/or research materials, please review your statements relating to the author contributions, and ensure you have specifically and accurately indicated the role(s) that these authors had in your study. You can update author roles in the Author Contributions section of the online submission form.

Please also include the following statement within your amended Funding Statement.

“The funder provided support in the form of salaries for authors [insert relevant initials], but did not have any additional role in the study design, data collection and analysis, decision to publish, or preparation of the manuscript. The specific roles of these authors are articulated in the ‘author contributions’ section.”

If your commercial affiliation did play a role in your study, please state and explain this role within your updated Funding Statement.

2. Please also provide an updated Competing Interests Statement declaring this commercial affiliation along with any other relevant declarations relating to employment, consultancy, patents, products in development, or marketed products, etc.

Within your Competing Interests Statement, please confirm that this commercial affiliation does not alter your adherence to all PLOS ONE policies on sharing data and materials by including the following statement: ""This does not alter our adherence to PLOS ONE policies on sharing data and materials.” (as detailed online in our guide for authors http://journals.plos.org/plosone/s/competing-interests) . If this adherence statement is not accurate and there are restrictions on sharing of data and/or materials, please state these. Please note that we cannot proceed with consideration of your article until this information has been declared.

Please include both an updated Funding Statement and Competing Interests Statement in your cover letter. We will change the online submission form on your behalf.

**Reply**

Thank you for the comment. Some of the authors, H.U., K.S., R.A., K.H., S.T., and M.I. are HOMER ION Co., Ltd. employees, however the authors believe there are no competing interests arising from this affiliation. This does not alter the authors' adherence to all the PLOS ONE policies on sharing data and materials. There are no planned patents or commercial products resulting from this work.

Funding and competing interests were added to the cover letter.

**Comment 8**

8. Please include your full ethics statement in the ‘Methods’ section of your manuscript file. In your statement, please include the full name of the IRB or ethics committee who approved or waived your study, as well as whether or not you obtained informed written or verbal consent. If consent was waived for your study, please include this information in your statement as well.

**Reply**

Thank you for the comment. The Ethics Review and approved No. for Animal Experimentation were added to the Animal section of Materials & Methods.

**Comment 9**

9. If the reviewer comments include a recommendation to cite specific previously published works, please review and evaluate these publications to determine whether they are relevant and should be cited. There is no requirement to cite these works unless the editor has indicated otherwise.

**Reply**

Thank you for the comment. The reviewers' comments did not recommend citing any specific published papers.

When additional citations were necessary for the manuscript, I cited papers of my own choosing as appropriate.

Reviewer #1 (Comments to the Author (Required)):

**Comment 1**

1. The introduction is redundant; it is unclear in L55-81 whether this study is an extension of your research or whether you are trying to establish a new experiment based on previous research. The aim of the study should be clearly indicated.

**Reply**

We greatly appreciate your valuable comments. We have revised the Introduction, manuscripts according to your comments.

**Comment 2**

2. A variety of factors can cause muscle atrophy, but the authors used the peripheral neuropathy model in this study. However, there is no research introduction on the use of this model. Please add this point to the Introduction section.

**Reply**

Thank you for the comment. A research introduction to the denervation model has been added to the Introduction.

**Comment 3**

3. The Methods and Materials section describes the animals as follows: "Acute stimulation experiment, chronic stimulation experiment, n=52." Could you please provide the specific number of animals in each group? Similarly, when stating that acute stimulation experiments were performed in three groups and chronic stimulation experiments were divided into four groups, the number of rats in each group should be specified.

**Reply**

Thank you for the comment. Added the specific number of animals in each group to Materials & Methods.

**Comment 4**

4. For Western blotting, is a loading control or total protein amount quantified at the same time to eliminate differences in protein expression between samples and the effects of manipulation during the experiment? The authors should add these to the Material & Methods section.

**Reply**

Thank you for the comment. In the Western blot, total protein was quantified using ponseau and normalized. I added this to the manuscript as per your comment.

**Comment 5**

5. The authors described “Stimulation intensity was preliminarily tested with a belt electrode and set to a minimum current run value of 3.0 mA (1.2 mA for twitch), which produced maximum torque with 60 Hz stimulation.” The authors should add the results of the preliminary investigation to Material & Methods section (or supplemental files).

**Reply**

We greatly appreciate your valuable comments. The results of a preliminary study on current strength are added to Figure 1b.

**Comment 6**

6. In “Acute response to electrical stimulation with belt electrodes” section, why did the authors examine the glycogen content and phosphorylated AMPK immediately after exercise and phosphorylated p70S6K in 6 h after exercise? Please provide a reason for the time to harvest the skeletal muscles. The authors did not assess the phosphorylated AMPK and p70S6K in TA. This investigation was titled “multiple muscle groups”, the lack of these results is a primary concern for this study.

**Reply**

We greatly appreciate your valuable comments. AMPK activation is observed during and immediately after exercise, and then rapidly returns to the basal level.( Am J Physiol 270: E299E304.) Phosphorylation of p70S6K (mTOR) gradually and significantly increased up to 6 hours after exercise cessation. Therefore, AMPK analysis was performed immediately after stimulation, and phosphorylation of p70S6K analysis was performed 6 hours later. We also performed the same experiments in TA muscles.

References were added to the manuscript.

**Comment 7**

7. After denervation, the sciatic nerve may sometimes reattach. To avoid this, the proximal and distal portions are often removed a few millimeters from the amputated area. Did the authors perform this procedure? Or, did they confirm that no reattachment occurred by dissection?

**Reply**

Thank you for the comment. The sciatic nerve was removed by a few millimeters. We have revised the Materials＆Methods manuscripts according to your comments.

**Comment 8**

8. Since the total RNA concentration differs from sample to sample, the amount of RNA contained in the RNA solution (5 μL) differs for each sample. Fortunately, the authors have determined the total RNA concentration by Nanodrop; please provide the total RNA concentration of each sample used for the 18S and 28S searches and show that there is no difference between groups.

**Reply**

Thank you for the comment. The RNA concentration was calculated as the amount of RNA per muscle weight and corrected for muscle weight. Each RNA concentration was added as supplementary information.

**Comment 9**

9. Regarding RT-PCR, how did the authors quantify Atrogin-1 and MuRF1 mRNA expression　? Did you use the internal control? If so, the primer sequences should be listed in Table 1.

16. In Figure 6, did the authors use the loading control (e.g., GAPDH)

**Reply**

Thank you for the comment. In this experiment,β-actin was used as an internal control. Concerning GAPDH, the glycolytic reaction is involved in carbohydrate metabolism in skeletal muscle research. Thus, it is difficult to use as a method for addressing muscle atrophy. Since the amount of ribosomes is reduced in atrophied muscles (Kotani, et al. J Appl Physiol.2022), 18S is not suitable for our experiment. These are the reasons why β-actin was selected in this study.

As per your comment, we added to Materials & Methods and added primer sequences to the Table.

**Comment 10**
10. It is difficult to confirm inter-individual variation in the mean±standard error. Please indicate the data as mean±standard deviation (SD). The authors should also add about the post-hoc tests. Furthermore, much data was listed as folds. Please re-describe all the data in mean±SD.

**Reply**

Thank you for the comment. Materials & Methods, Figures, and Figure legends have been revised. Post-hoc tests have also been added to Materials & Methods, and graphs have been revised to show raw data or divided values, except for Western blots, electrophoresis, and RT-PCR for chronic stimulation. Relative comparisons were performed for Western blots, electrophoresis, and RT-PCR for chronic stimulation.

**Comment 11**
11. In Figure 3, the band images of p-p70S6K and p-AMPK are too weak to confirm; please re-prepare the band images along with t-p70S6K and t-AMPK. Furthermore, two bands can be identified in p-p70S6K. Which of the two bands did you adopt?

**Reply**

Thank you very much for the comment. The band image in Figure 3 was re-prepared. The band of p-p70S6K was shown in cell signaling, and the upper band with high intensity was used.

**Comment 12**
12. In Figure 4, why did you separate the muscle wet weight into right and left sides? I suppose they should be combined. In addition to muscle wet weight, please add data on body weight and relative weight ratio.

**Reply**

Thank you for the comment. Data for muscle mass weight are presented separately to show that both left and right sides of the body had reduced muscle atrophy. Body weight, relative body weight ratio were added to Figure 4.

**Comment 13**
13. In Figure 5, which color indicates which muscle fiber type? Furthermore, since the scale bar is not listed, the sizes cannot be compared. Why is this data listed as a percentage while the muscle wet weight is listed as raw data? All data should be presented as raw data, not as ratios.

**Reply**

Thank you for the comment. The relationship between color and muscle fiber was added to Fig legends. Scale bars were added, and graphs of raw data were replaced.

**Comment 14**

14. Which muscles did the authors use for analyses of COXIV, 18S, 28S, Atrogin-1, and MuRF1? You must indicate all the data of the above parameters in TA and GAS.

**Reply**

Thank you for the comment. Data for COXIV, 18S, 28S, Atrogin-1, and MuRF1 were used in GAS, which showed a significant muscle atrophy suppression effect on muscle mass.

In addition, I added a note to the Fig legend Result to clarify which muscle group was used, as commented.

**Comment 15**
15. There is confusion between the terms “Murf1” and “MuRF1” (L355, 361). Please unify the descriptions.

**Reply**

Thank you for the comment. As you mentioned, the terminology was confusing. We have unified the notation.

**Comment 16**

16. In Figure 6, did the authors use the loading control (e.g., GAPDH)

**Reply**

Thank you for the comment. β-actin was used as a loading control. As you pointed out, this information was not included in the Materials & Methods section, so I added it to the Table, and the primer sequences were added.

**Comment 17**
17. The contents of L385-392 are duplicated in the Introduction section and are not necessary.

**Reply**

Thank you for the comment. We have revised the manuscripts according to your comments.

**Comment 18**
18. The authors described “muscle atrophy progresses from extracellular matrix degradation due to increased MMPs associated with decreased mitochondrial content.” (L410-412) I can’t understand this cascade. The author states that the degradation of muscle component proteins is a cause of muscle atrophy, and in this context, the degradation of the extracellular matrix (ECM) is a key factor. The primary function of MMPs is to degrade the ECM, and they are not directly involved in the degradation of muscle component proteins. This point should be described in more detail, and previous studies should be cited.

**Reply**

We appreciate your valuable comments. As commented, this paper does not examine effects on the extracellular matrix.

The effect of MMPs was deemed unnecessary and was removed from the description.

**Comment 19**
19. The authors described “twitch EMS increases mitochondrial mass and enzyme activity while suppressing muscle proteolytic signaling” in the Introduction section (L57-58). However, even though COXIV was significantly higher in DEN+CS than in DEN+60, there was no significant difference in 18S and 28S between the two groups. Furthermore, surprisingly, there was no significant difference in Atrogin-1 and MuRF1 between these two groups, despite significant differences in CSA for each muscle fiber type in GAS and TA. The author must add your idea on this discrepancy in the Discussion section.

**Reply**

Thank you for the comment. As per your comment, no discussion of the discrepancy was provided.

A discussion was added to Discussion.

**Comment 20**

20. Considering the title of this study, the authors likely aim to demonstrate the effectiveness of twitch and tetanus contractions on multiple skeletal muscles. This is understandable given that the study selected the TA (flexor) and GAS (extensor), which have distinct functional roles, as sample muscles. Additionally, since both skeletal muscles exhibit significant differences in muscle fiber size between their deep and superficial layers, the authors may have also wanted to investigate this point.

I read the discussion section expecting these points to be addressed, but unfortunately, there was not much discussion on the above topics. I would like you to reconsider the results and discussion regarding the following points.

1. Discuss whether any characteristic differences were found in the results of the tibialis anterior and gastrocnemius muscles between the acute and chronic experiments. Regardless of whether differences were found or not, discuss your findings in relation to previous studies.

2. Please discuss the differences in effects between the superficial and deep layers of the tibialis anterior and gastrocnemius muscles (if necessary, present the results separately for the superficial and deep layers). Based on the content of above 1, please discuss the effects of twitch and tetanus in the superficial and deep layers.

**Reply**

We appreciate your valuable comments. We have added a discussion to the Discussion section regarding the superficial and deep layers of the tibialis anterior and gastrocnemius muscles, as well as the results of acute and chronic tests.

Reviewer #2 (Comments to the Author (Required)):

**Comment 1**
1. This is a well-designed study combining twitch and tetanus stimulation to combat muscle atrophy. The idea is novel and valuable, especially for clinical applications in populations with limited mobility. However, please more clearly differentiate how your combined approach surpasses traditional methods using only tetanic stimulation, especially in terms of clinical scalability or mechanistic superiority.

**Reply**

We greatly appreciate your valuable comments. I agree with your comments and added them to Discussion for clinical significance and expansion.

**Comment 2**
2. The acute and chronic study arms are well constructed. Still, it would help to include a schematic diagram summarizing group allocation, stimulation protocols (e.g., duration, frequency, intensity), and outcome measurements.

**Reply**

Thank you for the comment. A schematic diagram has been added to Fig. 1 to show the electrical conditions in detail.

**Comment 3**

3. Auhors shown a good evidence that mixed stimulation enhances both mitochondrial and protein synthesis markers. Still, consider adding a discussion on possible molecular pathways that explain the additive/synergistic effects.

**Reply**

We appreciate your comments. I have added the discussion to molecular pathways that explain the additive/synergistic effects as per your comment.

**Comment 4**

4. While results are promising, some comparisons (e.g., between DEN+60 and DEN+CS) show only trends, not significant differences. Please clarify which results are statistically significant and which are not.

**Reply**

Thank you for the comment. As you indicated, it is difficult to tell which results are significant and which are not. We reviewed the entire paper and revised it to clarify significance and trends.

**Comment 5**

5. The figures are informative, but some (especially Western blot and CSA images) appear low-resolution. Please upload clearer images and ensure consistent formatting of figure legends.

**Reply**

Thank you for the comment. As per your comment, Fig and Fig legends have been modified.

**Comment 6**

6. Include exact p-values wherever possible and define the number of animals (n) per group for each analysis in both the text and figure legends.

**Reply**

Thank you for the comment. Revised p-values and number of animals (n) in the text and figures.

**Comment 7**

7. The manuscript reads well overall, but a few sections (especially the Results and Discussion) would benefit from language polishing for clarity and flow.

**Reply**

Thank you for the comment. We have reviewed the manuscript polishing for clarity and flow in accordance with your suggestion.

**Comment 8**

8. Author mention future use in postoperative or elderly patients. It would be great to briefly explain how this method could be implemented practically in clinical or rehabilitation settings.

**Reply**

Thank you for the comment. Added Discussion on clinical significance.

**Comment 9**

9. Please discuss limitations such as the short duration (7 days) of chronic stimulation and whether longer-term studies or human trials are being considered.

**Reply**

Thank you for the comment. As per your comment, this paper is an evaluation at chronic stimulation (7 days), so it is unclear how long-term intervention and timing of initiation of stimulation will affect on muscle atrophy. Limitations of this study have been added to the Discussion section.

**Comment 10**

10. Maintain consistent terminology (e.g., use "TA" and "GAS" uniformly, and define all abbreviations on first use) to avoid confusion for non-specialist readers.

**Reply**

Thank you for the comment. Modified manuscript to ensure consistent definitions of abbreviations and terminology.

**Reviewer #3 (Comments to the Author (Required)):**

**Comment 1**

Introduction

Describe in detail the molecular mechanisms by which combined stimulation therapy was predicted to more effectively inhibit denervation sarcopenia compared to twitch or tetanus stimulation alone at the planning stage of the study.

**Reply**

Thank you for the comment. A comparison with single stimuli, including molecular mechanisms, was added to the Introduction.

**Comment 2**

Methods

Please provide more detailed information regarding the rationale for selecting the stimulation parameters used in this study, including the frequency and temporal distribution of the combined stimuli.

**Reply**

Thank you for the comment. As per your comment, I have added to Materials & Methods and Figures the reasons for the selection of stimulus parameters.

**Comment 3**

Results

Muscle wet weight may have been affected by each rat’s variability, such as body weight. Please provide the muscle wet weight and body weight data for each rat at the time of sacrifice.

**Reply**

Thank you for the comment. For rats, body weight at the time of delivery was specified, and care was taken to ensure that there was no variation in the test. As you indicated, body weight is important, so we added data on body weight and relative body weight ratio in Figure 4.

**Comment 4**

Histological Evaluation

In the CSA graphs, the black dots appear to represent ratios; however, the definition of the comparison target is unclear. Do these values represent, within each muscle, the total cross-sectional area of each muscle fiber type, or the average cross-sectional area per fiber type?

To better visualize fiber size changes, I recommend adding muscle fiber CSA distribution plots, as shown in your previous paper.

The composition of muscle fiber types (fast-twitch and slow-twitch) has been reported to change in sarcopenia. And electrical stimulation affects the composition of muscle fiber type (Shi H, J Neuroeng Rehabil, 2023). Please provide supplemental data on fiber type composition (e.g., number and proportion of each fiber type) in each muscle examined.

**Reply**

Thank you for your comment. As you pointed out, we have added explanations of the data to the legend of the figure to improve the clarity of the data. In addition, we have added a distribution graph of muscle fiber cross-sectional area (CSA) to Fig. and fiber type composition to the supplement data.

**Comment 5**

Western Blotting

In the supplementary data, it is unclear which bands correspond to the same samples between p-p70S6K and t-p70S6K, as well as between p-AMPK and t-AMPK. The correspondence between these bands should be clearly indicated. Additionally, loading controls such as GAPDH or β-actin are not shown, making it difficult to confirm equal protein loading. Please include these controls.

**Reply**

Thank you for the comment. To clarify the sample correspondence in total and phosphorylation, Fig data were modified to include the sample in each band. The intensity of each band was corrected for total protein content. The correction was also added to the Figure legands.

**Comment 6**

Discussion

Although the description of the effects and mechanisms of B-SES is informative, some of this content may be more appropriately presented in the Introduction section.

**Reply**

Thank you for the comment. As per your comment, the effects and mechanisms of B-SES are described in the Introduction, removed from the Discussion.

**Comment 7**

The absence of interference between twitch and tetanus stimulation is a significant finding. Please expand the discussion on the molecular mechanisms that may underlie this result.

The combination of twitch and tetanus stimulation more suppressed the expression of muscle degradation markers. It would be informative if the authors could discuss the possible molecular pathways involved in this mechanism.

**Reply**

Thank you for the comment. As per your comment, there was little mention of molecular mechanisms. We have added to Discussion that there was no interference with twitch and tetanus contraction in this study and more suppression of expression of muscle degradation markers and molecular mechanisms.
