# Supplementary material for: Mixed twitch and tetanus electrical stimulation via belt-electrode effectively attenuates denervation-induced muscle atrophy
Source: PLoS One. 2025 Oct 15;20(10):e0334691. doi: 10.1371/journal.pone.0334691 (PMC12527172; doi:10.1371/journal.pone.0334691)
Supplement: Attachment — Submitted filename: Manuscript Number PONE-D-25-28377R1.docx [file pone.0334691.s004.docx]

**Manuscript Number PONE-D-25-28377R1**

The manuscript “Mixed twitch and tetanus electrical stimulation via belt-electrode effectively attenuates denervation-induced muscle atrophy” presents a novel strategy where combined twitch and tetanus stimulation more effectively preserves skeletal muscle mass than tetanus alone. The study highlights that this approach enhances both mitochondrial biogenesis and protein synthesis, addressing key pathways in denervation-induced atrophy. Importantly, the authors carefully revised the manuscript to address all editorial and reviewer concerns, including clarifying methodology, improving figures, ensuring statistical rigor, and expanding discussion on mechanisms and clinical applications

Decision: Based on its novelty, translational potential, and thorough revisions, I recommend acceptance of this manuscript for publication in PLOS ONE.
